# Toxicity of aqueous extracts of *Ilex paraguariensis* A.St.‐Hil. about *Euphorbia heterophylla* L.

**DOI:** 10.1002/ps.70701

**Published:** 2026-03-05

**Authors:** Tamara Alberton da Silva, Ikram Bashir, Ana Caroline Giacomin, Jéssica Adriane Barth, Marina Chiapperin, Fabieli Zanotelli de Oliveira, Camila Thaís Scheibler, Fernanda Bruxel, Elisete Maria de Freitas

**Affiliations:** ^1^ Curso de Ciências Biológicas Universidade do Vale do Taquari – Univates Lajeado Brazil; ^2^ Laboratório de Botânica Universidade do Vale do Taquari – Univates Lajeado Brazil

**Keywords:** allelopathy, bioherbicide, decoction, infusion, weed plant, yerba mate

## Abstract

**BACKGROUND:**

The escalating challenge of herbicide‐resistant weeds, exemplified by *Euphorbia heterophylla* L. (wild poinsettia), threatens agricultural sustainability in Brazil. Overreliance on synthetic herbicides has led to environmental degradation and increased production costs, necessitating eco‐friendly alternatives.

**RESULTS:**

This study explores the phytotoxic potential of aqueous extracts (decoction and infusion) from *Ilex paraguariensis* A.St.‐Hil. (yerba mate) as a bioherbicide against *E. heterophylla*. High‐performance liquid chromatography (HPLC) identified nine major compounds in aqueous extracts (decoction and infusion), with caffeine and neochlorogenic acid being the most abundant. *In vitro* assays demonstrated that both decoction and infusion extracts at concentrations 4% and 6% completely inhibited seed germination and seedling formation of *E. heterophylla*, with lower concentrations (2%) significantly reducing germination speed and increasing mean germination time. Glasshouse experiments revealed mild to moderate leaf damage (scales 2–3) from 4% extracts, without affecting height or true leaf emergence. Field trials indicated temporal stability in development, with extracts promoting slight biomass increases (root/shoot ratio) and modulating antioxidants and pigments (chlorophyll A, cholorphyll B, carotenoids), showing positive correlations with growth traits and less severity than glyphosate.

**CONCLUSION:**

Aqueous extracts of *I. paraguariensis* exhibit strong allelopathic potential against *E. heterophylla*, particularly during germination, offering a biodegradable bioherbicide option for integrated pest management. While less toxic to mature plants, their selectivity and scalability warrant further field optimization to enhance sustainable agriculture, reducing environmental impacts and herbicide resistance. © 2026 The Author(s). *Pest Management Science* published by John Wiley & Sons Ltd on behalf of Society of Chemical Industry.

## Graphical abstract

Aqueous extracts of *Ilex paraguariensis* A.St.‐Hil. strongly inhibit *Euphorbia heterophylla* L. germination and early seedling growth, but show mild phytotoxicity on mature plants, paradoxically promoting biomass via antioxidants.

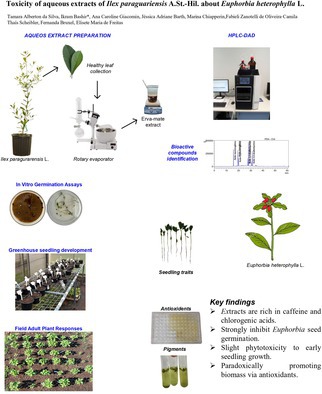

## 1 INTRODUCTION

Weed control remains a critical challenge threatening agricultural sustainability and productivity in Brazil. *Euphorbia heterophylla* L., commonly known as wild poinsettia, is a highly problematic weed in Brazilian croplands. Its resilient seeds persist in the soil for years, competing aggressively with crops and reducing yields.^1^ Over the past decade, this weed has developed widespread resistance to synthetic herbicides, especially acetolactate synthase (ALS) inhibitors, particularly in key agricultural states like Paraná and Rio Grande do Sul. This resistance has increased control costs and complicated management efforts.^2^

Despite Brazil's remarkable success in expanding agricultural production, generating billions of reais annually, the heavy reliance on synthetic herbicides has imposed severe economic and environmental costs. Overuse has driven widespread resistance (exemplified by *E. heterophylla*), soil degradation, water contamination, and biodiversity loss, jeopardizing the long‐term viability of agroecosystems.^3^, ^4^, ^5^ Economically, resistance forces farmers to apply higher doses or switch to more expensive products, eroding profit margins in a highly competitive global market.^6^ These challenges have heightened the need for sustainable alternatives that maintain productivity while protecting the environment.

Allelopathy, where plants release biochemicals that inhibit neighboring species, offers a promising natural approach to weed control through bioherbicides.^7^ Yerba mate (*Ilex paraguariensis* A.St.‐Hil.), locally known as erva‐mate, is a culturally iconic plant in southern Brazil. Consumed as chimarrão; a hot infusion shared communally from a gourd (cuia) using a metal straw (bomba), it symbolizes friendship, hospitality, and regional identity in states like Rio Grande do Sul, Paraná, and Santa Catarina.^8^, ^9^ Brazil leads global yerba mate production, with cultivation centered in the south, supporting family farms, thousands of jobs, and substantial revenue from domestic consumption and exports.^10^

Aqueous extracts from *I. paraguariensis* have demonstrated allelopathic effects, inhibiting weeds such as *Conyza bonariensis* and suggesting potential as a bioherbicide.^11^ Rich in secondary metabolites like saponins, polyphenols, and methylxanthines (including caffeine), these extracts disrupt seed germination and seedling growth in target species.^12^, ^13^ Unlike synthetic herbicides, plant‐derived bioherbicides are biodegradable, leave minimal residues, and reduce environmental contamination.^14^ Compounds such as caffeine, rutin, and quercetin in yerba mate extracts exhibit phytotoxic activity with potentially lower non‐target impacts.^11^

In Brazil, agriculture is dominated by soybean, corn, and sugarcane crops. Weeds in these fields frequently cause yield loses exceeding 30%.^3^, ^15^ Bioherbicides could manage resistant weeds like *E. heterophylla*, slow the evolution of new resistance, preserve export revenues, and safeguard aquatic ecosystems and public health by reducing chemical runoff.^16^, ^17^, ^18^, ^19^ Aligning with global trends toward sustainable farming, allelopathy is increasingly integrated into weed management strategies, enhancing soil microbial diversity and ecological balance.^20^, ^21^, ^22^, ^23^, ^24^

Leveraging yerba mate, a culturally embedded crop, could transform local by‐products into valuable bioherbicides, supporting agroforestry and intercropping systems while providing economic benefits, such as reduced dependence on imported chemicals.^3^ This approach could also help mitigate the environmental burden of Brazil's approximately 70 000 tons of annual pesticide use, much of which contributes to pollution.^25^ Challenges remain, including the need for further research on extract selectivity toward crops, production scalability, and field efficacy against *E. heterophylla*. Preliminary studies show that higher concentrations (e.g., 1.0% decoction) significantly reduce germination and seedling vigor in target weeds.^11^

This study evaluates the allelopathic effects of *I. paraguariensis* aqueous extracts on *E. heterophylla*, aiming to contribute to sustainable weed management in Brazil. By harnessing a native, culturally significant plant, it seeks to advance a more resilient, environmentally friendly agricultural future.

## 2 MATERIALS AND METHODS

### 2.1 Collection of botanical material and preparation of aqueous leaf extracts from *I. paraguariensis*

Leaves of *I. paraguariensis* were harvested from an organic cultivation site located in the municipality of Ilópolis, Rio Grande do Sul, Brazil, (28°54′38.3″ S, 52°06′23.8″ W). A fertile branch was collected in the area of occurrence of the herb and deposited in the Herbarium of Vale do Taquari – HVAT, under registration HVAT 7607. Concurrently, seeds of *E. heterophylla* were gathered from an urban area in the municipality of Lajeado, Rio Grande do Sul, Brazil (29°26′44.4″ S and 51°56′12.5″ W). The leaves underwent a selection process, during which those exhibiting visible damage, lichen growth, or signs of insect infestation were discarded. The remaining leaves were dehydrated in an oven with air circulation at 50 °C until they achieved a consistent dry mass. Following dehydration, the leaves were pulverized using a willey‐type knife mill (Willey Macro‐Tn650/1 model, Tecnal, Piracicaba, Brazil). The resulting material was then utilized to prepare aqueous extracts through decoction and infusion methods.^11^ For this study, the pure leaf decoction (LD) and leaf infusion (LI) extracts were diluted to concentrations of 2% (LD2, LI2), 4% (LD4, LI4), and 6% (LD6, LI6), two control treatments were also included: a negative control (CT−), consisting solely of purified water, and a positive control (CT+), comprising a 2% glyphosate herbicide solution as specified in the product label, bringing the total to eight treatments shown in Table 1.

**Table 1:** Concentrations of aqueos extracts of *Ilex paraguariensis* A.St.‐Hil. obtained by leaf decoction (LD), leaf infusion (LI) and control treatment used in different experiments.

Treatment	Code	Exp‐1	Exp‐2	Exp‐3
Purified water	CT−	**×**	**×**	**×**
Glyphosate 2%	CT+	**×**	**×**	**×**
Leaf decoction 2%	LD2	**×**		
Leaf decoction 4%	LD4	**×**	**×**	**×**
Leaf decoction 6%	LD6	**×**		
Leaf infusion 2%	LI2	**×**		
Leaf infusion 4%	LI4	**×**	**×**	**×**
Leaf infusion 6%	LI6	**×**		

*Note*:  Exp‐1: experiment 1, *in vitro* germination of *Euphorbia heterophylla* L.; Exp‐2: experiment 2, glasshouse seedling development of *E. heterophylla*; Exp‐3: experiment 3, young plant of *E. heterophylla* development in field test.

### 2.2 Phytochemical characterization of the extracts of *I. paraguariensis*

The extracts were analyzed for the presence of 19 phenolic compounds including 3,4‐dicaffeoylquinic acid, 3,5‐dicaffeoylquinic acid, 4,5‐dicaffeoylquinic acid, apigenin, caffeic acid, caffeine, catechin, chlorogenic acid, cryptochlorogenic acid, myricetin, neochlorogenic acid, oleanolic acid, *p*‐coumaric acid, quercetin, rutin, syringic acid, theobromine and ursolic acid based using high‐Performance Liquid Chromatography‐Mass Spectrometry (HPLC‐MS), LC‐20AT model (Shimadzu, Kyoto, Japan) with diode array detector (DAD). The identification of the compounds was based on the comparison of the area of the peaks and retention times of 19 compounds, with analytical reference standards for each compound using linear equations obtained from calibration curves. The analysis was carried out at the Instrumental Center of the Technological Center for Food Research and Production – CTPPA – of Universidade do Vale do Taquari (Univates), Lajeado, RS, Brazil. The presence of the compounds was confirmed by the analysis of the retention times, compared to those obtained for the reference analytical standards of each compound, and their quantification was performed by calculating the areas of the peaks obtained in the chromatogram, plotted in the linear equation obtained from the calibration curves.

### 2.3 Phytotoxicity of *I. paraguariensis* extracts

#### 2.3.1 *In vitro* assays

To evaluate the effects on the germination and seedling formation, Petri dishes were used with eight treatments (CT+, CT−, LD2, LD4, LD6, LI2, LI4, LI6) (Table 1). Each Petri dish contained three sheets of germitest paper that were surface sterilized for 20 min in a sterile ultraviolet (UV) light hood. Ten seeds of *E. heterophylla* were then placed on the germitest paper, and 8.0 mL of the respective extract concentration and control treatments were added. The Petri dishes were sealed with Parafilm and incubated in a growth chamber under a 16‐h photoperiod, at a temperature of 25 °C, a light intensity of 446 lx, in a completely randomized experimental design.

Germination and seedling formation were monitored every 24 h over an 11‐day period. Seeds were considered germinated when the radicle length reached or exceeded 0.5 mm. Seedlings were identified by the presence of at least one true leaf. Based on daily observations, the following parameters were calculated^11^: germination percentage (GP), mean germination time (MGT), speed germination index (SGI), seedling formation percentage (SFP), mean seedling formation time (MSFT), and the ratio of seedlings formed to seeds germinated (*S/G*).

The GP was determined by dividing the total number of germinated seeds per replicate by the total number of seeds used. The MGT represented the average number of days required for germination in each treatment replicate. The SGI was calculated using the equation: SGI = (G1/N1) + (G2/N2) + … + (G*n*/N*n*),^26^ where G1 is the number of seeds germinated at the first count, N1 is the number of hours elapsed until the first count, G2 is the number of seeds germinated at the second count, N2 is the number of hours elapsed until the second count, and so forth. The SFP was calculated as the number of seedlings formed per replicate relative to the number of germinated seeds. The MSFT was derived from the average time in days for seedling formation. The *S/G* ratio was determined by dividing the total number of germinated seeds by the total number of seedlings formed.

#### 2.3.2 Early development of seedlings of *E. heterophylla* in a glasshouse

To assess the phytotoxic effects of the extracts on the early development of seedlings in a glasshouse, 13‐day‐old *E. heterophylla* individuals were used. These seedlings had been germinated in plastic pots containing organo‐mineral substrate and exhibited two cotyledonary leaves. Based on the results obtained from the germination bioassay, four treatments (CT+, CT−, LD4, LI4) were used (Table 1) with four replicates of four plants (16 plants per treatment). Using a manual sprayer, 15 mL of each treatment was applied to each plant. Following application, the plants were placed in a glasshouse and arranged in a completely randomized experimental design. Randomization was performed by assigning numbers to each pot, and four treatments were applied by generating random number equal to total number of pots.

**Figure 1:**
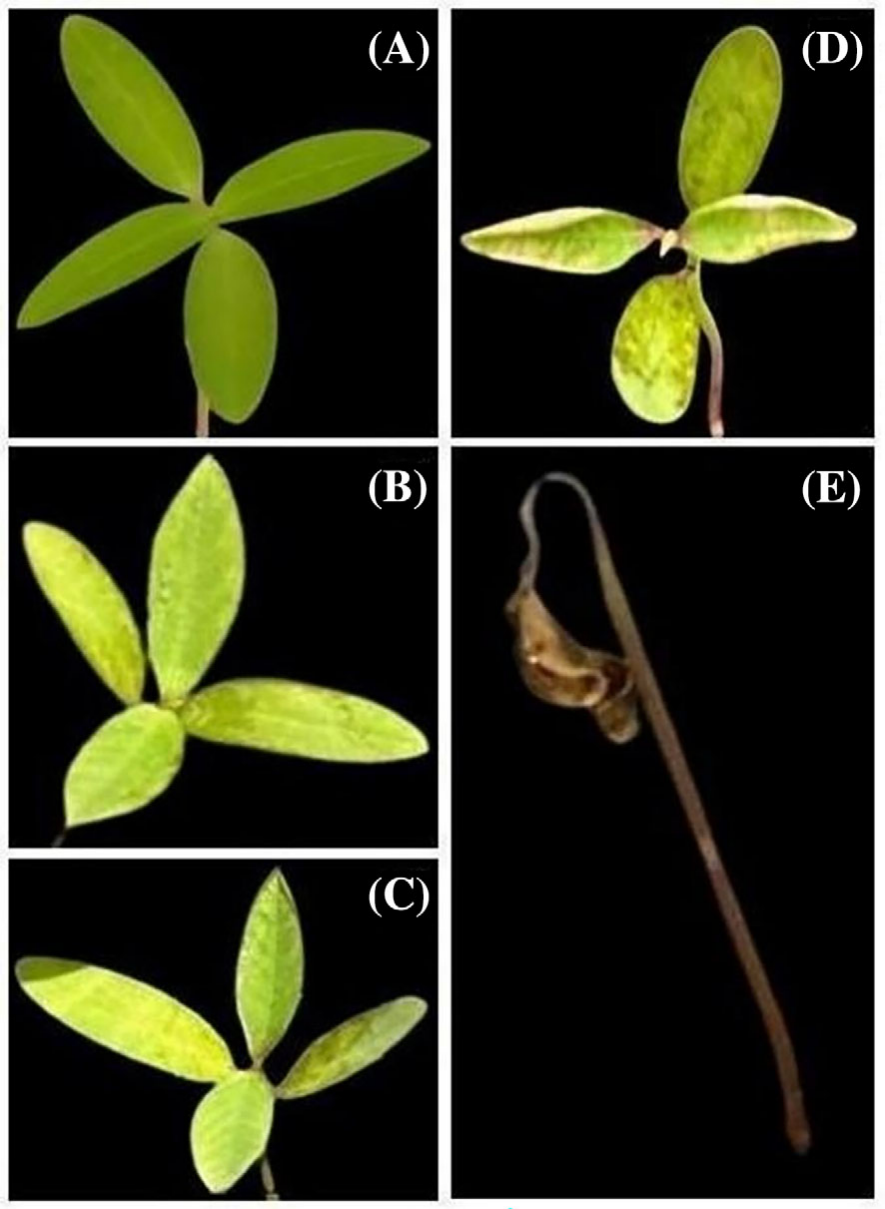
Degradation scale from 0 to 4, being (A) scale 0 for intact leaves (undamaged and very green) of *Euphorbia heterophylla* L., (B) scale 1 for light damage (only dark or slightly yellowish edges), (C) scale 2 for medium damage (spots all over the leaf, yellowing or starting the wilting process), (D) scale 3 for leaves with greater damage (spots and growth inhibition or leaves in advanced senescence) and (E) scale 4 for fatal damage (death).

The monitoring and evaluation of the seedlings early development were conducted over a period of 12 days. Observations were made every 24 h for the first 7 days and every 48 h thereafter, with the aim of assessing the formation of the first true leaf and any changes in leaf coloration. At the conclusion of the 12‐day observation period, a leaf damage assessment was performed using a scale ranging from 0 to 4: 0 indicated intact leaves (undamaged and vibrantly green), 1 denoted mild damage (only dark or slightly yellowed edges), 2 represented moderate damage (yellowed spots across the leaf or the onset of wilting), 3 indicated severe damage (spots and growth inhibition or leaves in advanced senescence), and 4 signified fatal damage (plant death) (Fig. 1).

#### 2.3.3 Phytotoxic effect under field conditions on young plants of *E. heterophylla*

To assess the phytotoxic effects of the extracts on *E. heterophylla* seedling development under field conditions, two experimental plots were established in the Botany Laboratory field area (29°27′00″ S, 51°56′38″ W) at the Univates. *E. heterophylla* seeds were manually sown at a depth of 2 cm, with three replications per plot. Both plots received the same treatments (CT−, CT+, LD4, LI4) as those used in the glasshouse experiment (Table 1). In the first plot, treatments were applied to 20‐day‐old plants. Leaf samples were collected 1 and 7 days after treatment application. In the second plot, three uniform plants per replication were selected at the cotyledon stage for treatment application. Seedling development was evaluated 1, 3, and 7 days after treatment by recording the total number of true leaves and assessing leaf damage using a 0–4 scale (0 = intact leaves, 4 = dead seedling; Fig. 1). After 7 days, shoot and root lengths were measured by carefully uprooting the plants and manually recording their lengths (in centimeters).

The contents of chlorophyll A, chlorophyll B and carotenoid were determined.^27^ Briefly, 0.01 g samples were added in test tubes with 7 mL of dimethyl sulfoxide (DMSO) (with 5% calcium carbonate (CaCO_3_)) and it was made sure that leaf sample was fully immersed in the solvent. Test tubes were placed in a water bath at 65 °C for 30 min, until the leaf disc is transparent. The test tubes were kept in the dark until they reached room temperature. Then, 250 μL of solvent were pipetted in microplates Cuvette 96 wells for spectrophotometer determinations at three wavelengths 480 nm, 649 nm and 665 nm. The theories of chlorophyll A, chlorophyll B, and total carotenoids were calculated using formulas given in Eqns ((1), (2), (3))–((1), (2), (3)).

(1) $$\text{Chlorophyll}\;A\;\left(C_{A}\right)=\left(12.19\times A_{665}\right)-\left(3.45\times A_{649}\right)$$

(2) $$\text{Chlorophyll}\;B\;\left(C_{B}\right)=\left(21.99\times A_{649}\right)-\left(5.32\times A_{665}\right)$$

(3) $$\text{Carotinoid}\;\left(C\right)=\frac{\left(1000\times A_{480}\right)-\left(2.14\times C_{A}\right)-\left(70.16\times C_{B}\right)}{220}$$

To determine the activity of the antioxidant enzymes, superoxide dismutase (SOD), catalase (CAT) and ascorbate peroxidase (APX), there was initially an extraction in which 0.2 g of the sample was macerated in a porcelain mortar, in the presence of liquid nitrogen. Then, 375 μL of phosphate buffer 100 mm (pH 7.8), 15 μL of EDTA (ethylenediaminetetraacetic acid) 0.1 mm, 75 μL of ascorbic acid 10 mm and 1035 μL of ultrapure water were added and centrifuged at 13 000 rpm at 4 °C for 20 min. From that extract, the SOD activity was determined from inhibition in the reduction of NBT (*p*‐nitro blue tetrazolium) by the enzymatic extract, avoiding the formation of chromophore.^28^ In this essay, a SOD enzymatic activity unit was considered a necessary enzyme to obtain 50% of the inhibition to reduce NBT by SOD contained in the enzyme extract. For the reaction 10 μL of the enzyme extract was added in a test tube containing 100 μL of potassium phosphate buffer 50 mm (pH 7.8), 40 μL of methionine 14 mm, 2 μL of EDTA 0.1 μm, 31 μL of ultrapure water, 15 μL of NBT 75 μm and 2 μL of riboflavin 2 μm. After that, the tubes were incubated in a 15 W fluorescent lamp for 10 min, then the absorbance was read at 560 nm. For calculation, the white of the reaction was considered as the tubes that did not contain extract, exposed and not exposed to the light. The activity was determined by the calculation of the extract that inhibits 50% of the reaction of NBT and expressed in units (U) per gram of fresh weight of leaf sample (U g^−1^ FW).

The APX^29^ and CAT^30^ activity was determined by the consumption of hydrogen peroxide (H_2_O_2_). A 10 μL aliquot of the extract was added to a 100 μL potassium phosphate buffer 100 mm (pH 7.0), 70 μL of ultrapure water and 20 μL of H_2_O_2_ 12.5 mm. The absorbance was read using a spectrophotometer (Spectramax *i*3) microplate reader (Molecular Devices, San Jose, CA, USA) in the wavelength of 240 nm. In the case of APX activity, 10 μL aliquot of the extract was added to a 150 μL potassium phosphate buffer 37.5 mm (pH 7.0), 20 μL of ascorbic acid 0.25 mm and 20 μL of H_2_O_2_ 5 mm. The absorbance was read at the wavelength of 290 nm. Both the CAT and APX activity was done for 180 s, with readings in intervals of 15 s. The activities of total extract were determined by the calculation of the amount of extract that reduced the reading of absorbance and expressed in millimolar concentration per gram of fresh weight per minute (mm g^−1^ FW min^−1^).

### 2.4 Data analysis

The data obtained in the bioassay evaluations were tested for normality and homoscedasticity by using Shapiro–Wilk and Levene test, respectively. Later, data was submitted to analysis of variance (ANOVA) and Tukey's test. All the analysis were performed using the statistical program R.^29^ All figures were developed using the R package ggplot2.

## 3 RESULTS

### 3.1 *I. paraguariensis* aqueous extracts phytochemical composition

**Figure 2:**
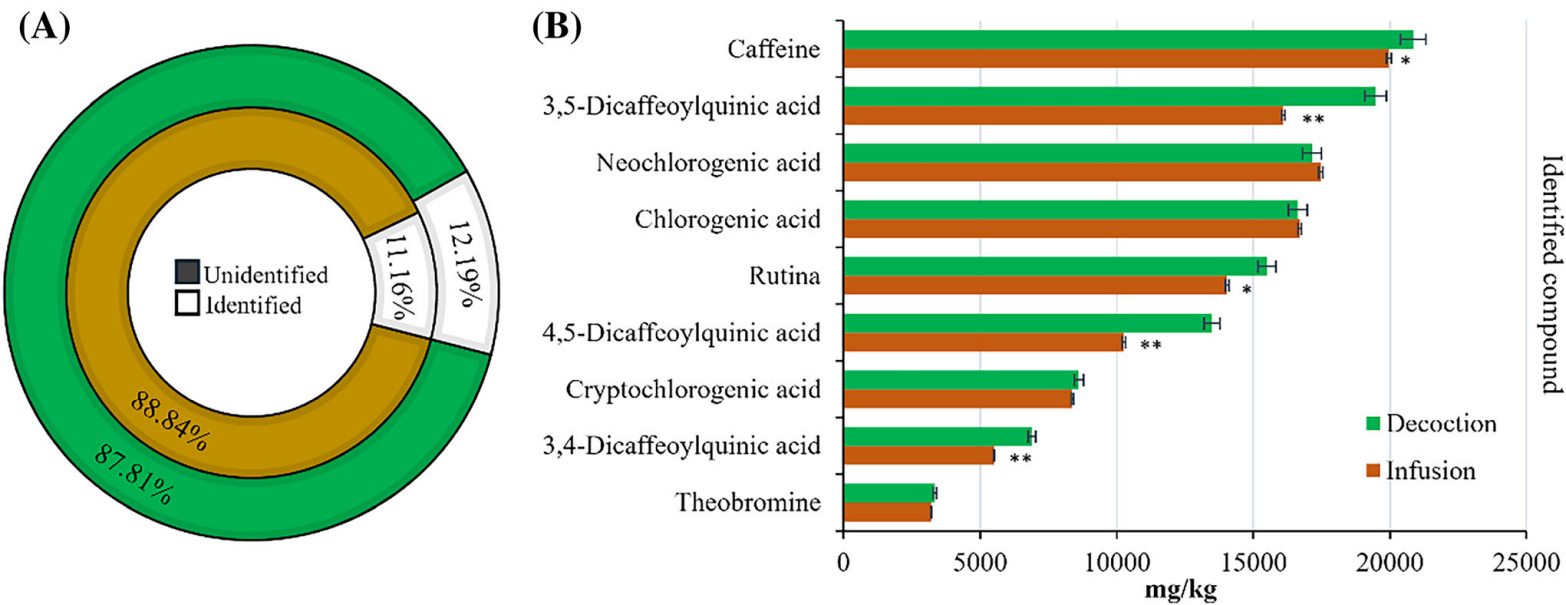
(A) Circular plot represents the percentage of total compound identified (with no color filling) and unidentified (with solid color filling) in *Ilex paraguariensis* A.St.‐Hill. plant decoction and infusion extracts. (B) Bar plot represent the detection limits of each compound identified in the respective decoction and infusion extracts (mg kg^−1^ ± standard deviation) by high‐performance liquid chromatography–mass spectrometry (HPLC–MS). *P < 0.05; **P < 0.01.

The HPLC‐MS using DAD analysis identified and quantified nine major compounds among the 19 tested: theobromine, neochlorogenic acid, chlorogenic acid, cryptochlorogenic acid, 3,4‐dicafeoylquinic acid, 3,5‐dicafeoylquinic acid, 4,5‐dicafeoylquinic acid, caffeine and rutin (Fig. 2 and Supporting Information, Table Figure S1). Among the identified compounds, caffeine and neochlorogenic acid were present in greater quantity. The comparison between the extraction methods showed that the infusion and decoction resulted in statistically significant differences in the chemical composition of the extracts, and the decoction process presented a higher number of compounds such as caffeine. In the total composition of the extracts, 12.19% of the extract obtained by decoction and 11.15% by infusion can be quantified.

### 3.2 *I. paraguariensis* extracts affect the *in vitro* germination

**Figure 3:**
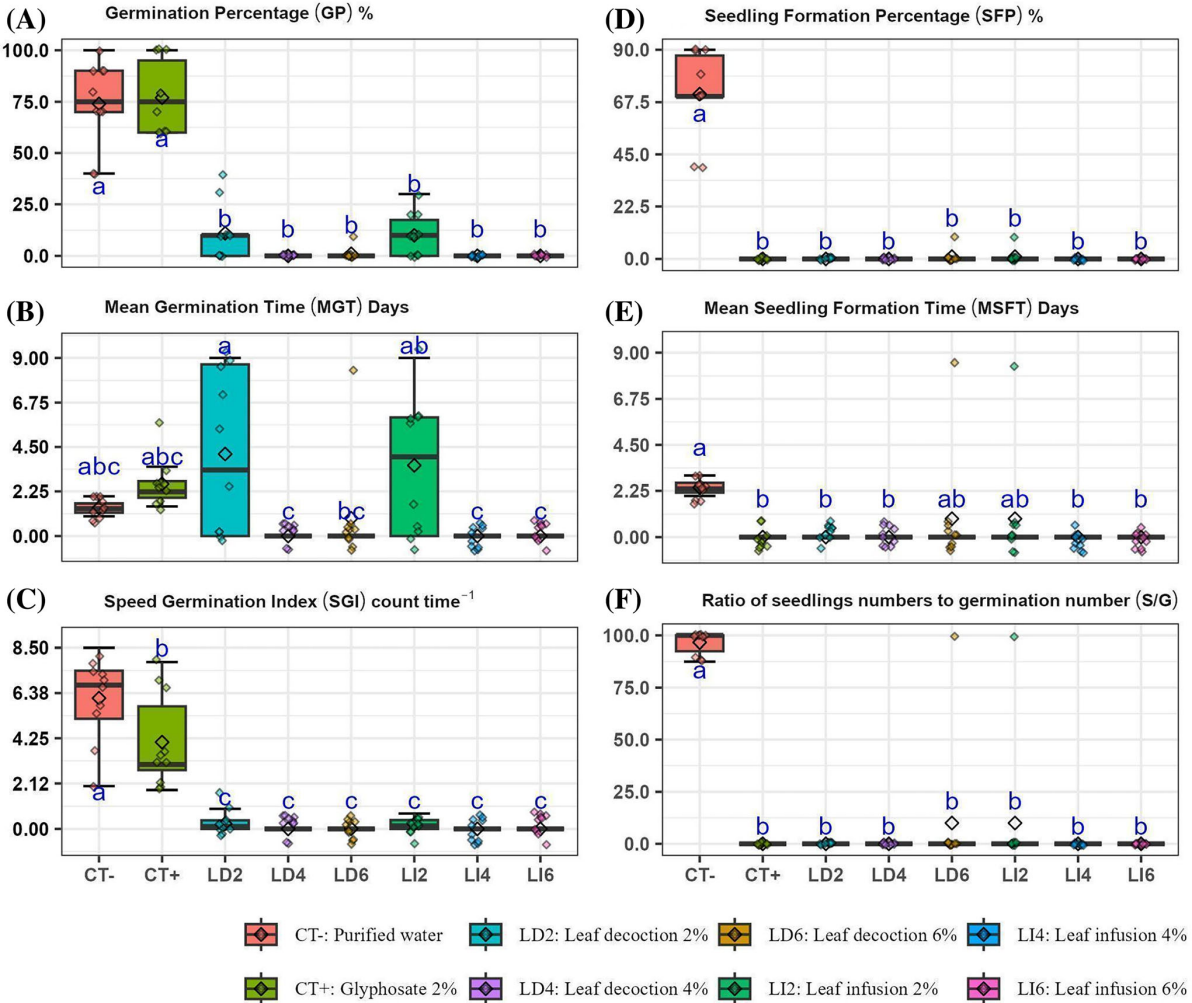
Mean and standard deviation of the germination percentage (GP), mean germination time (MGT), speed germination index (SGI), seedling formation percentage (SFP), mean seedling formation time (MSFT) and the ratio between the number of seedlings formed and the number of germinated seeds (*S/G*) of *Euphorbia heterophylla* L. exposed to synthetic herbicide glyphosate (CT+) and aqueous extracts by leaf decoction (LD) and leaf infusion (LI) of *Ilex paraguariensis* A.St.‐Hil. leaves, submitted to analysis of variance (ANOVA) and Tukey's test.

All treatments with the extracts showed low GP values or zero, differing statistically (*P* < 0.0001) from the CT− and CT+ treatments, which were statistically equal. Among the treatments with extracts, germination was observed in LD2 and LI2, but with much lower average than the controls (11% and 10%, respectively). In LD6 there was only one seed germinated (Fig. 3(A)).

For the MGT (Fig. 2(B)), the LD2 and LI2 treatments showed higher MGT (4.14 ± 3.97 days and 3.57 ± 3.32 days, respectively) in relation to the two controls, not differing from them. The two treatments differed from the other treatments with extracts, except for LD6 (*P* < 0.0001). In the LD4, LD6, LI4 and LI6 treatments, there was no germination in most replications, keeping the average time close to zero and, for this reason, statistically, they did not differ from the controls.

Regarding the SGI (Fig. 3(C)), all treatments with the extracts showed mean values low or equal to zero, in relation to the two controls. CT− had the highest SGI and differed from CT+ (*P* < 0.0001). When we consider the variables related to seedling formation, there was no seedling formation in any of the treatments with the extracts, which also occurred with CT+. Therefore, all of them differed from the CT− (*P* < 0.0001).

### 3.3 Phytotoxic effect on the initial development of seedlings

**Figure 4:**
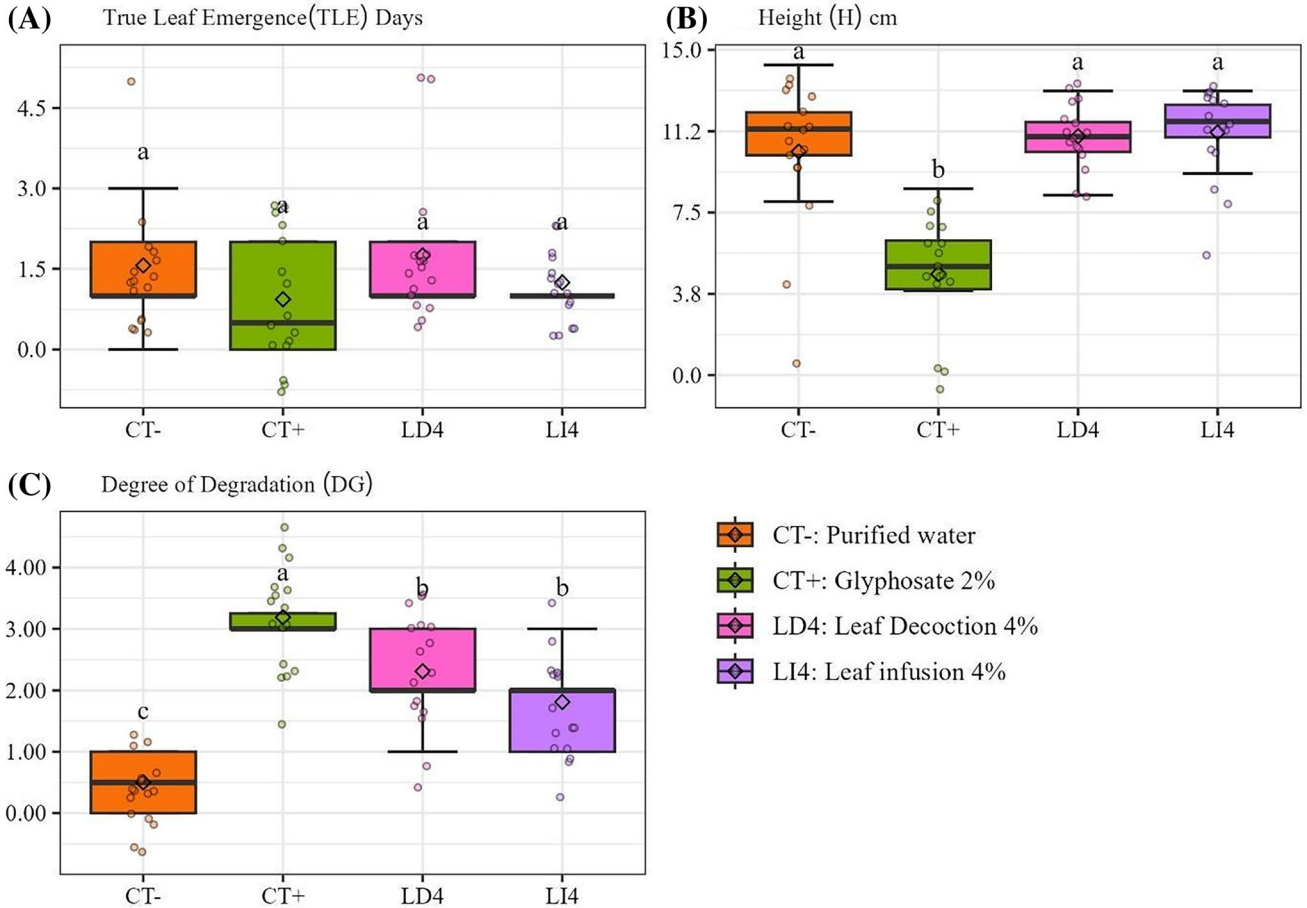
Number of true leaf emergence (TLE), height (*H*) and degree of degradation (DG) of seedlings of *Euphorbia heterophylla* L. in early development exposed to the purified water (CT−), synthetic herbicide glyphosate (CT+), 4% aqueous extracts by decoction (LD4) and 4% infusion of leaves (LI4) of *Ilex paraguariensis* A.St.‐Hil.

The treatments did not differ from each other in terms of the number of days for the emergence of the first true leaf (Fig. 4(A)), although in CT+ there was a 2‐day delay in the emergence of the first true leaf in practically half of the replicates (43.75%) and inhibited the emergence in the other half (50%). Regarding height (*H*), the treatments with the application of extracts (LD4 and LI4) and CT− did not differ statistically (*P* < 0.0001) (Table 2) from each other but differed from CT+ (Fig. 4(B)).

**Table 2:** Analysis of variance (ANOVA), number of true leaf emergence (TLE), height (*H*) and degree of degradation (DG) of *Euphorbia heterophylla* L. exposed to aqueous extracts of *Ilex paraguariensis* A.St.‐Hil.

Variables	Treatment (*P* valor)	CV (%)	General mean
TLE	<0.0001	13.20	1.52
*H*	<0.0001	23.30	9.30
DG	<0.0001	32.18	1.95

*Note*: CV, coefficient of variation.

Regarding the degree of degradation (DG), the treatments with the application of the extracts (LD4 and LI4) differed statistically from the two controls. Both caused damage to the leaves, but these were lower than the damage caused by CT+ (68.75% of the replicates had damage classified as scale 3 and 25% were classified as scale 4). In LD4, 87.5% of the repetitions showed some degree of damage, half on scale 2 and the other half on scale 3. In LI4, 43.75% of the repetitions were classified on scale 2 and 18.75% as major damage grade 3 (Fig. 4(C)). In the CT−, 50% of the repetitions were classified on a scale of 0 (intact = no damage) and the other 50% presented some mild damage, classified on a scale of 1.

### 3.4 Temporal effects of *I. paraguariensis* aqueous extracts on *E. heterophylla* plant development

**Figure 5:**
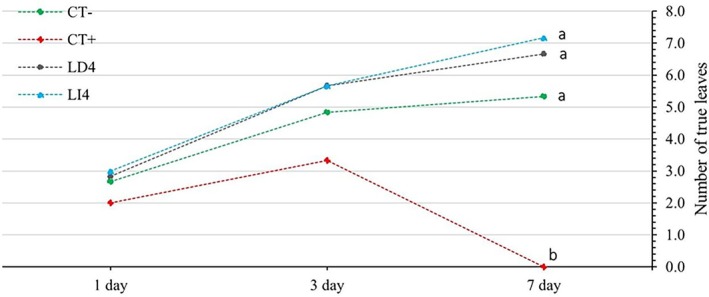
Temporal response of the phytotoxic effect on the number of true leaves developed of *Euphorbia heterophylla* L. after applying different treatments of *Ilex paraguariensis* A.St.‐Hil. extract from decoction 4% (LD4), infusion 4% (LI4), compared with two controls of pure water (CT−) and glyphosate 2% (CT+) under field conditions. The lowercase letter represents the significant differences based on Tukey's test among different treatments.

The line graph (Fig. 5) illustrates the number of true leaves developed in plants under four treatments – CT− (purified water), CT+ (glyphosate 2%), LD4 (leaf decoction 4%), and LI4 (leaf infusion 4%) – over a period of 1, 3, and 7 days. On day 1, all treatments start with a similar number of true leaves, approximately two, showing no significant differences. By day 3, CT−, LD4, and LI4 exhibit a steady increase to around 4–5 true leaves, while CT+ peaks at around four leaves but shows a slight decline compared to the others. By day 7, CT−, LD4, and LI4 continue to rise, reaching approximately 6–7 true leaves, and indicating statistically similar and robust leaf development. In stark contrast, CT+ plummets to around one true leaf, demonstrating a significant decline and highlighting the detrimental effect of glyphosate on leaf development over time. Overall, the results suggest that *I. paraguariensis* extracts (LD4, LI4) support consistent leaf growth and did not show any toxic effect over the period monitored compared to glyphosate (CT+) which severely inhibited true leaf development by day 7.

### 3.5 *I. paraguariensis* extracts promote biomass production in *E. heterophylla*

**Figure 6:**
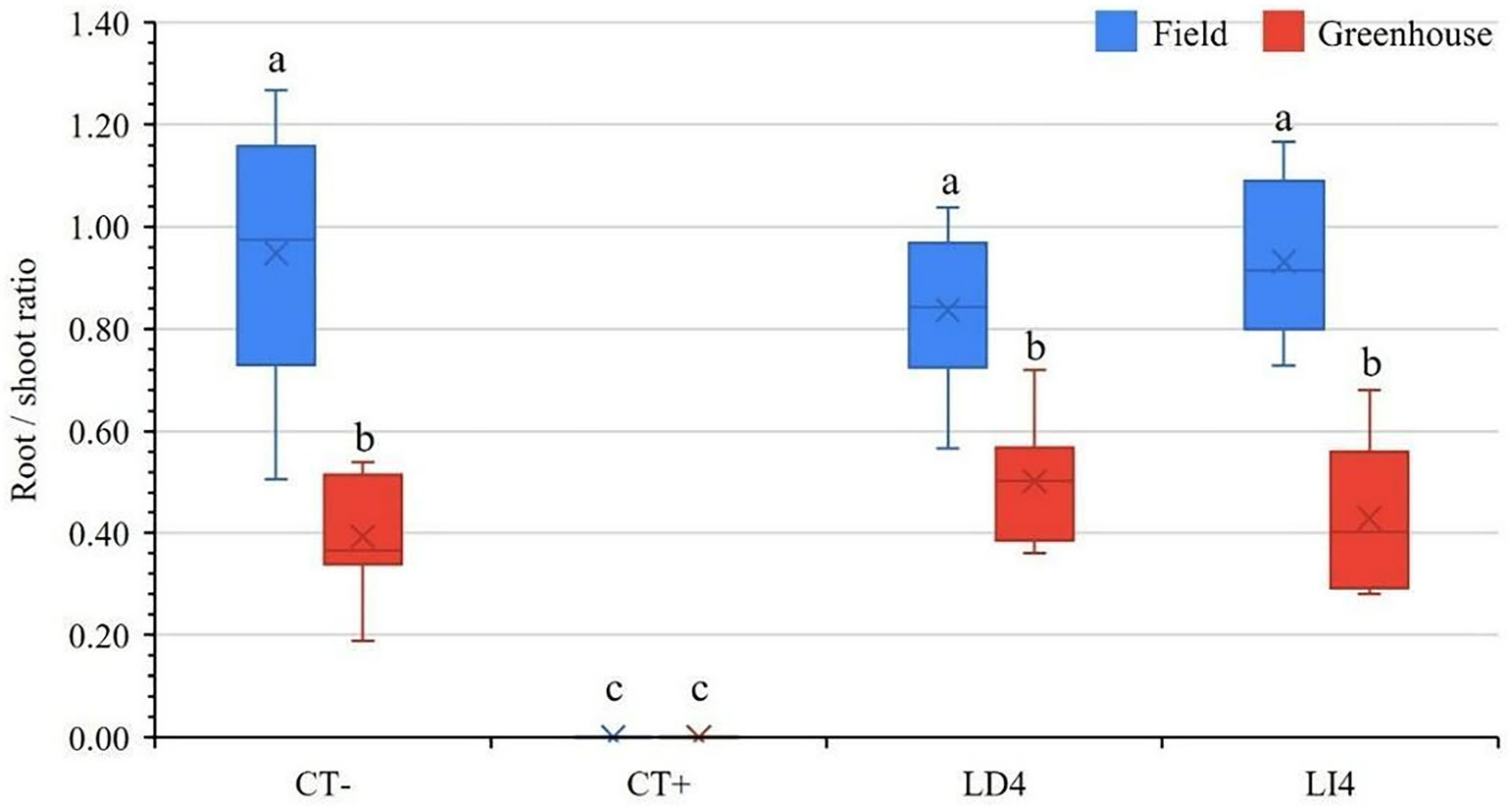
Phytotoxic effect on the root/shoot length ratio of *Euphorbia heterophylla* L. after applying different treatments of *Ilex paraguariensis* A.St.‐Hil. extract from decoction 4% (LD4), infusion 4% (LI4), compared with two controls of pure water (CT−) and glyphosate 2% (CT+) under glasshouse and field conditions. The lowercase letter represents the significant differences based on Tukey's test among different treatments and growth conditions.

Figure 6 illustrates the root/shoot ratio of *E. heterophylla* plants treated with *I. paraguariensis* various extracts under field and glasshouse conditions, with treatments including CT− (purified water), CT+ (glyphosate 2%), LD4 (leaf decoction 4%), and LI4 (leaf infusion 4%). The root/shoot ratio, a key indicator of biomass allocation and plant stress response, varied significantly across treatments. For CT− (control), both field and glasshouse conditions exhibited a baseline root/shoot ratio of approximately 0.4–0.9, indicating normal growth without phytotoxic influence. In contrast, CT+ (glyphosate) markedly reduced to death of the plants across both environments, reflecting a strong phytotoxic effect consistent with glyphosate (CT+) known herbicidal action. Treatments LD4 and LI4, derived from leaf decoction and infusion respectively, showed higher ratios in the field (0.9–1.0) compared to the glasshouse (0.3–0.4), suggesting a very slight phytotoxic effect. However, under field conditions, biomass in terms of root/shoot ratio was higher as compared to glasshouse conditions.

### 3.6 *I. paraguariensis* extract application modulated the physiological features in foliage of *E. heterophylla*

**Figure 7:**
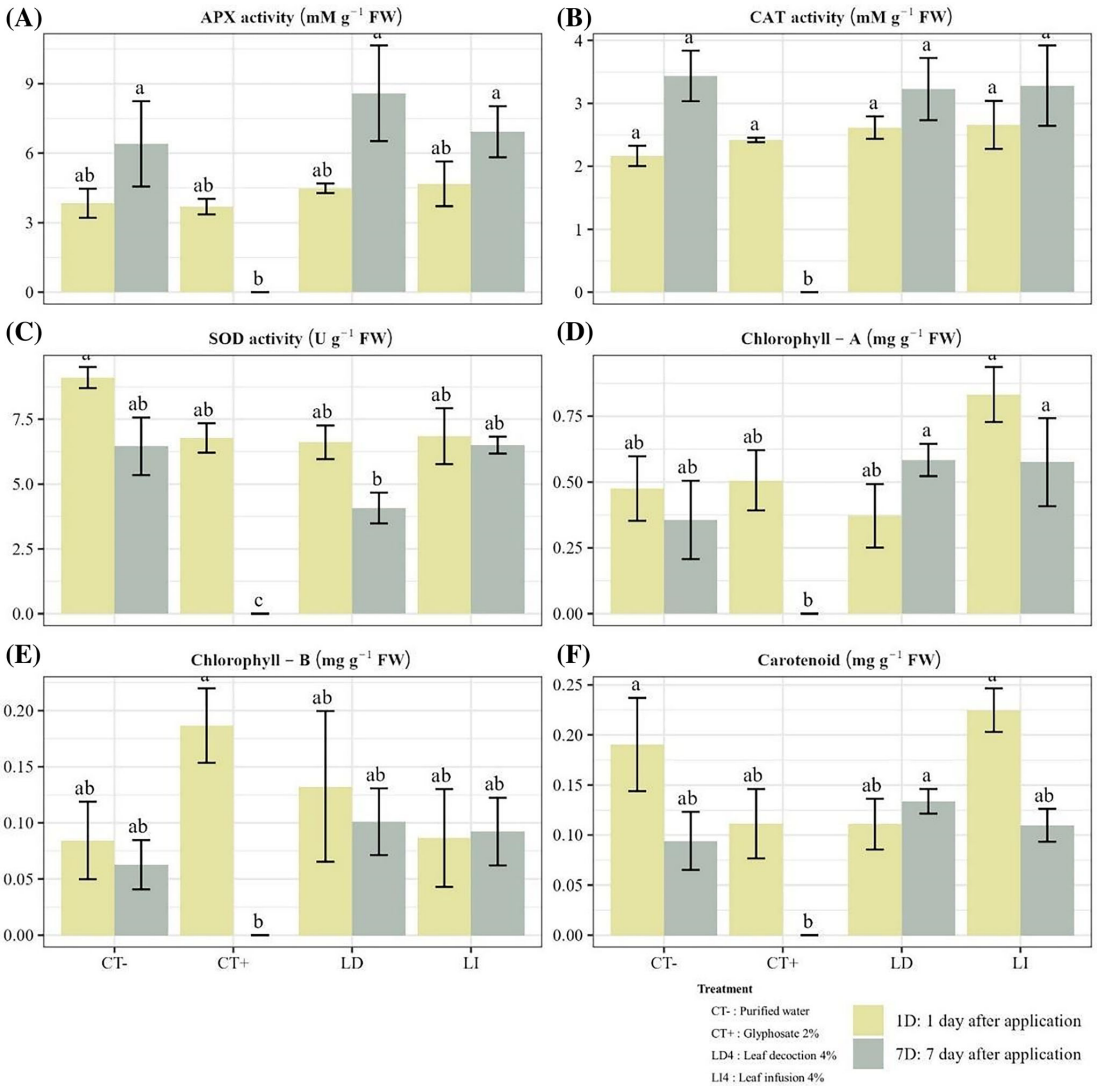
Effect of different treatment of *Ilex paraguariensis* A.St.‐Hil. extract on (a) ascorbate peroxidase (APX) activity, (b) catalase (CAT) activity, (c) superoxide dismutase (SOD) activity, (d) chlorophyll A, (e) chlorophyll B and (f) carotenoid content (E) of *Euphorbia heterophylla* L. plants grown in field conditions measured after 1 day and 7 days of application. The bar diagrams represent the mean values of three replicates. Corresponding error bars represents standard deviation of three replicates. The lowercase letter represents the significant difference at 0.05% among the treatments and days after different variables observed based on Tukey's test.

To assess the phytotoxic effect of the *I. paragureinsis* on *E. heterophylla*, we performed biochemical analysis including antioxidants such as APX, CAT, SOD and photosynthetic pigment analysis through DMSO method including chlorophyll A, chlorophyll B and carotenoid shown after 1 and 7 days of application of extracts treatment under field conditions shown in Fig. 7(A)–(F). The negative control (CT−, pure water) exhibited moderate APX and CAT activities, with a notable increase in APX at 7 days after application (6.40 *versus* 3.84), while SOD activity decreased (6.46 *versus* 9.11). Pigment levels (cholorphyll A, cholorphyll B, carotenoid) in CT− declined over time, indicating natural degradation. The positive control (CT+, glyphosate 2%) showed severe phytotoxicity, with all measured variables (antioxidants and pigments) dropping to zero after 7 days of application, reflecting complete plant stress and death. In contrast, the leaf decoction (LD4) and leaf infusion (LI4) treatments demonstrated milder phytotoxic effects. LD treatment increased APX activity significantly after 7 days of application (8.58 *versus* 4.48), suggesting enhanced oxidative stress response, while CAT remained stable and SOD decreased. LI4 treatment showed a similar trend, with APX rising moderately (6.93 *versus* 4.68) and stable CAT and SOD levels. Pigment contents in LD4 and LI4 varied: chlorophyll A increased in LD4 after 7 days of application (0.58 *versus* 0.37) but decreased in LI4 (0.58 *versus* 0.83), while chlorophyll B and carotenoid showed slight fluctuations. These findings indicate that yerba‐mate extracts induce oxidative stress and pigment alterations, but their phytotoxic effects are less severe than glyphosate, with LD4 potentially stimulating certain stress responses over time.

## 4 DISCUSSION

The germination and initial seedling development tests conducted in this study demonstrated the phytotoxic effects of infusion and decoction extracts from *I. paraguariensis* leaves on *E. heterophylla*. However, in contrast to similar studies,^11^ where the phytotoxic potential of aqueous *I. paraguariensis* extracts on *C. bonariensis* were evaluated and stronger inhibitory effects from the decoction extract at 1% concentration on germination were reported, we employed higher concentrations. This choice was made because parts of our experiments were performed under glasshouse and field conditions, where various uncontrolled environmental factors could influence the results. In our study, however, the lowest concentration tested (2%) for both extracts did not fully inhibit germination. In contrast, the intermediate concentration (4%) of both extracts achieved complete inhibition, clearly demonstrating phytotoxicity on *E. heterophylla* seeds. A similar study on the allelopathic effects of aqueous extracts from dry *Lantana camara* L. leaves on *Daucus carota* L. (carrot) confirmed their phytotoxicity.^30^ However, effective concentrations differed: germination was reduced only at high levels (7.5–10%), compared to reduction at our lowest concentration (2%) and complete inhibition at 4%. These differences likely stem from extract preparation methods, which may be the reason why toxic action occurred at the lower concentrations of our study. Furthermore, just as in the study of *C. bonariensis* ^11^ showed that the extracts obtained through the process of decoction of dried leaves were more toxic when compared to extracts obtained by infusion of dry leaves, this study also confirmed such evidence.

In addition to reducing GP values at the lowest concentration (2%) of both extracts, there was an increase in MGT and a consequent decrease in the SGI. These findings indicate that the extracts exhibit toxicity even at low concentrations, adversely affecting the germination process. The prolonged germination time increases the likelihood of negative impacts on subsequent survival stages.^31^ The SGI values further confirmed the toxicity of the extract treatments on *E. heterophylla* germination, showing significantly lower means compared to controls, and even lower than those observed with CT+. The impaired seedling vigor (reduced SGI) can lead to stunted growth, heightened sensitivity to stress and predation, diminished competitive ability for resources, and overall compromised development.^32^ Moreover, at the intermediate concentration (4%), both extracts caused such intense inhibition that all evaluated variables were zero, reinforcing the similar findings^11^ while suggesting that these higher concentrations may ensure more consistent and effective phytotoxic outcomes.

The results revealed that the infusion and decoction extracts of *I. paraguariensis* exhibited stronger phytotoxicity during germination than on established plants. While plant height and time to true leaf formation remained unaffected compared to CT+, the extracts induced leaf damage, primarily necrosis, similar in intensity to that caused by glyphosate, positioning it intermediately between the positive and negative controls. This leaf necrosis aligns with glyphosate's mode of action, which is absorbed through leaves and roots, translocated systemically, and inhibits essential amino acid synthesis, leading to gradual plant death.^33^ A comparable mechanism may involve compounds in the *I. paraguariensis* extracts, as leaf necrosis is a classic symptom of phytotoxic inhibition.^34^ Supporting this, necrosis in all weed seedlings treated with Myrtaceae leaf extracts, exceeding the damage caused by their herbicide control,^35^ while similar necrosis and plant death was also observed in young plants of *C. bonariensis* treated with *I. paraguariensis* extracts.^11^

Under field conditions, where *E. heterophylla* germinates and develops more vigorously due to favorable natural factors, the extracts showed limited phytotoxicity on adult plants. Biomass measurements (root/shoot ratio) after 12 days revealed no significant reduction compared to the CT− notably, the 4% infusion treatment even increased biomass relative to its decoction counterpart. This absence of strong inhibition on mature plants echoes low caffeine doses improved growth in lentils.^36^ Given that caffeine comprised only approximately 2% of identified compounds in our extracts, its concentration was likely insufficient for marked toxicity in field‐grown adult plants.

Chemical characterization confirmed the extracts' richness in bioactive compounds (Fig. 2), with caffeine and neochlorogenic acid predominant in both infusion and decoction, consistent with Tamura *et al*.,^37^ who identified these as major components in yerba mate extracts. The predominance of caffeine and neochlorogenic acid in our extracts aligns with various findings reporting these as the main compounds in various yerba mate‐based beverages, with concentrations varying according to preparation methods.^38^, ^39^ This consistency across studies reinforces that the core phytochemical profile of *I. paraguariensis* remains stable despite differences in extraction techniques and environmental factors. However, quantitative variations highlight that infusion and decoction are not equally efficient in extracting bioactive compounds, potentially affecting their phytotoxic efficacy in different applications.

Plant secondary metabolites confer adaptive advantages through allelopathy,^40^ making them valuable for agroecological weed management. The observed herbicidal potential of *I. paraguariensis* extracts evident in their toxicity to germination, seedling development, and young plants likely stems from the combined or individual action of compounds such as caffeine, rutin, and quercetin.^11^ Further research is needed to isolate these compounds from infusion and decoction extracts and evaluate their specific phytotoxic effects.

Analyzing plant growth parameters is crucial for understanding performance and productivity, as it reveals adaptive strategies in resource‐limited environments. In this study, we quantified photosynthetic pigments and antioxidant levels in *E. heterophylla* to assess physiological responses to *I. paraguariensis* extracts and confirm phytotoxicity. Photosynthetic pigments, such as chlorophylls and carotenoids, are vital for synthesizing organic compounds^41^ and typically decline under abiotic stress. Here, pigment concentrations increased under LI4, followed by LD4, indicating minimal stress‐induced degradation.^42^ Notably, biomass (root/shoot ratio) positively correlated with chlorophyll A and carotenoids but negatively with chlorophyll B (Supporting Information, Fig. Figure S1). Plants enhance antioxidant defenses to combat oxidative stress, primarily through enzymes like SOD, APX, and CAT.^43^ SOD acts as the first defense by converting superoxide to H_2_O_2_,^44^ while APX and CAT scavenge H_2_O_2_.^45^ Elevated enzyme activity often signals redox imbalance under stress.

In our study, application of *I. paraguariensis* extracts at 4% concentration (LD4 and LI4) elicited a significant but moderate increase in antioxidant enzyme activities (APX, CAT, and SOD), insufficient to cause terminal plant damage but indicative of a mild phytotoxic stress response that ultimately favored *E. heterophylla* development under field conditions. This aligns with hormetic effects observed in other systems, where low dose allelochemicals or plant extracts enhance antioxidant defenses and stimulate growth; for instance, a Brassica water extract at 2% concentration boosted wheat biomass and antioxidant enzymes (including SOD and CAT), with positive correlations between enzyme activities and growth traits.^46^ Similarly, pre‐sowing caffeine treatment (a key compound in yerba mate) maximized wheat seedling biomass increases while elevating SOD, CAT, and APX activities, showing strong positive correlations with biomass and growth parameters.^47^ Endogenous caffeine in rice likewise enhanced SOD, CAT, and APX levels, maintaining biomass and chlorophyll under water deficit without severe oxidative damage.^48^ These findings reinforce our observation of positive correlations between biomass traits and antioxidant enzymes in extract‐treated plants, substantiating that such applications on adult plants can promote development rather than hinder it.

## 5 CONCLUSION

This study provides valuable insights into the phytotoxic effects of *I. paraguariensis* extracts, offering a promising approach to managing one of Brazil's most invasive agricultural weeds, *E. heterophylla*. The research highlights the rich phytochemical profile of *I. paraguariensis* leaves, particularly bioactive compounds like caffeine, chlorogenic acid, and neochlorogenic acid, confirming existing literature. By comparing infusion and decoction extraction methods, the study found significant differences in chemical composition, with decoction proving more effective for extracting compounds like caffeine, despite both methods preserving bioactivity. While the findings underscore the plant's chemical diversity, many compounds remain unidentified, suggesting opportunities for further exploration. The extracts showed remarkable potential as a pre‐germination bioherbicide, with low doses (LD4 and LI4) effectively suppressing *E. heterophylla* germination when integrated with standard agronomic practices. Surprisingly, field experiments revealed that *I. paraguariensis* extracts also act as a growth stimulant for mature plants, potentially reducing weed competition while enhancing crop development. This dual functionality makes it a compelling candidate for sustainable agriculture. However, further research is needed to understand the bioherbicide's oxidative stress pathways in various species to minimize crop harm and to identify cell defensins and kinases activated by its toxicity. These efforts could lead to a comprehensive product for controlling invasive plants throughout the crop life cycle, revolutionizing eco‐friendly weed management and crop productivity.

## FUNDING INFORMATION

Secretaria de Inovação, Ciência e Tecnologia do Rio Grande do Sul, Brasil (Termo de Colaboração: SICT 08/2022; Processo PROA: 22/2500‐0000205‐3).

## CONFLICT OF INTEREST

The authors declare no conflicts of interest.

## AUTHOR CONTRIBUTIONS

Conceptualization: TAS, IB, EMF; methodology: TAS, IB, EMF; validation, formal analysis: IB; resources: EMF; data curation: TAS, IB, ACG, MC, JAB, FZO and FB; writing – original draft preparation: TAS, IB, ACG; writing – review and editing: TAS, IB; supervision: EMF; project administration: EMF. All authors have read and agreed to the published version of the manuscript.

## Supporting information


**Figure S1.** Correlation among physiological traits and root/shoot ratio of *Euphorbia heterophyll* L. measured under pure water (CT−), glyphosate 2% (CT+), leaf decoction 4% (LD4), leaf infusion 4% (LI4) *Ilex paraguriensis* A.St.‐Hil. and average of all treatment for root/shoot ratio (x̄)
**Table S1**. Identification and quantification of the active compounds (mg/kg ± SD) of different extracts obtained from the leaves of *Ilex paraguariensis* A.St.‐Hil. by HPLC–MS. *P ≤ 0.05; **P ≤ 0.01; ***P ≤ 0.001.

## Data Availability

Data is available on request.
