# Supplementary material for: Toxicity of aqueous extracts of Ilex paraguariensis A.St.‐Hil. about Euphorbia heterophylla L
Source: Pest Manag Sci. 2026 Mar 5;82(7):6196–206. doi: 10.1002/ps.70701 (PMC13240690; doi:10.1002/ps.70701)
Supplement: Supplementary file 1 — Figure S1. Correlation among physiological traits and root/shoot ratio of Euphorbia heterophyll L. measured under pure water (CT−), glyphosate 2% (CT+), leaf decoction 4% (LD4), leaf infusion 4% (LI4) Ilex paraguriensis A.St.‐Hil. and average of all treatment for root/shoot ratio (x̄) Table S1. Identification and quantification of the active compounds (mg/kg ± SD) of different extracts obtained from the leaves of Ilex paraguariensis A.St.‐Hil. by HPLC–MS. *P ≤ 0.05; **P ≤ 0.01; ***P ≤ 0.001. [file PS-82-6196-s001.docx]

**Supplementary Data:**


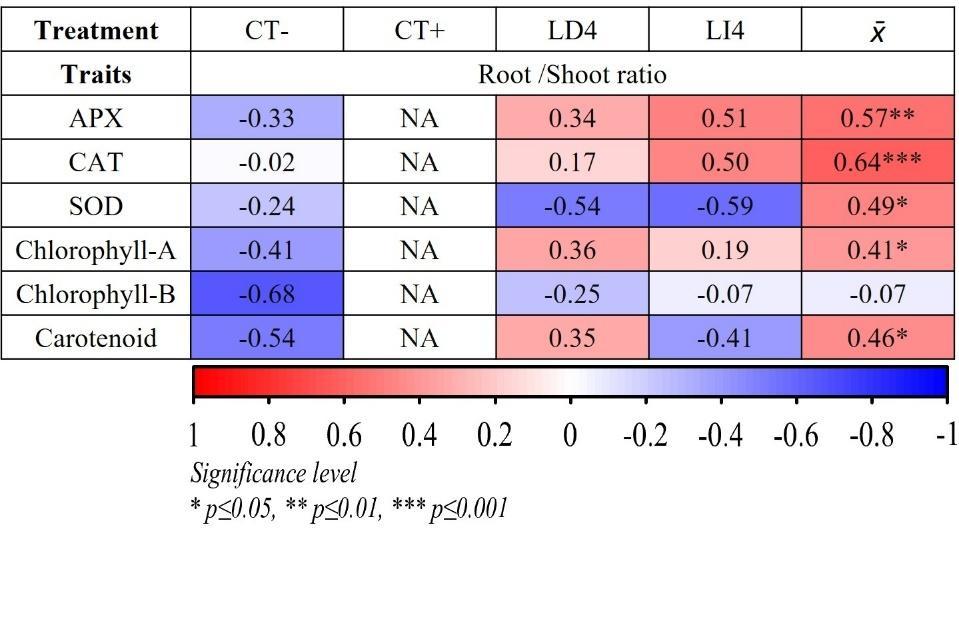


**Figure S. 1:** Correlation among physiological traits and root / shoot ratio of Euphorbia heterophyll L. measured under pure water (CT-), glyphosate 2% (CT+), leaf decoction 4% (LD4), leaf infusion 4% (LI4) Ilex paraguriensis A. St.-Hil. and average of all treatment for root / shoot ratio (x̄).

**Table S. 1:** Identification and quantification of the active compounds (mg/kg ± SD) of different extracts obtained from the leaves of Ilex paraguariensis by HPLC/MS. *p≤0.05; **p≤0.01; ***p≤0.001.

| **Active Compound** | **Retention time** | **Area** | **Infusion** | **Decoction** |
| --- | --- | --- | --- | --- |
| Theobromine | 14.87 | 1032569 | 3200.07 ± 5.57 | 3339.17 ± 72.90 |
| Neochlorogenic acid | 15.79 | 2579651 | 17464.73 ± 77.82 | 17143.02 ± 347.79 |
| Chlorogenic acid | 19.35 | 2479781 | 16700.81 ± 69.49 | 16629.14 ± 344.64 |
| Cryptochlorogenic acid | 20.02 | 1340920 | 8376.40 ± 40.04 | 8610.05 ± 179.30 |
| Caffeine | 20.67 | 7728587 | 19956.92 ± 83.51 | 20855.77 ± 460.16 |
| 3,4-Dicaffeoylquinic acid | 27.61 | 1219721 | 5508.53 ± 22.92 | 6887.92 ± 142.39 |
| 3,5-Dicaffeoylquinic acid | 28.06 | 3921259 | 16090.08 ± 58.11 | 19472.63 ± 409.8 |
| Rutin | 30.71 | 2873462 | 14035.70 ± 56.69 | 15492.98 ± 338.61 |
| 4,5-Dicaffeoylquinic acid | 31.22 | 2706284 | 10252.25 ± 69.17 | 13477.40 ± 289.71 |
